# Using a hospital admission survey to estimate the burden of influenza‐associated severe acute respiratory infection in one province of Cambodia—methods used and lessons learned

**DOI:** 10.1111/irv.12489

**Published:** 2018-02-16

**Authors:** Rebekah J. Stewart, Sovann Ly, Borann Sar, Vanra Ieng, Seng Heng, Kheng Sim, Chiedza Machingaidze, Katherine Roguski, Erica Dueger, Ann Moen, Reiko Tsuyuoka, A. Danielle Iuliano

**Affiliations:** ^1^ Epidemic Intelligence Service Centers for Disease Control and Prevention Atlanta GA USA; ^2^ Influenza Division National Center for Immunization and Respiratory Diseases Centers for Disease Control and Prevention Atlanta GA USA; ^3^ Communicable Disease Control Department Ministry of Health Phnom Penh Cambodia; ^4^ Influenza Program United States Centers for Disease Control and Prevention Phnom Penh Cambodia; ^5^ Emerging Disease Surveillance and Response World Health Organization Phnom Penh Cambodia; ^6^ Emerging Disease Surveillance and Response World Health Organization Regional Office for the Western Pacific Manila Philippines

**Keywords:** Cambodia, hospitalization, influenza, severe acute respiratory infection, surveillance

## Abstract

**Background:**

Understanding the burden of influenza‐associated severe acute respiratory infection (SARI) is important for setting national influenza surveillance and vaccine priorities. Estimating influenza‐associated SARI rates requires hospital‐based surveillance data and a population‐based denominator, which can be challenging to determine.

**Objectives:**

We present an application of the World Health Organization's recently developed manual (WHO Manual) including hospital admission survey (HAS) methods for estimating the burden of influenza‐associated SARI, with lessons learned to help others calculate similar estimates.

**Methods:**

Using an existing SARI surveillance platform in Cambodia, we counted influenza‐associated SARI cases during 2015 at one sentinel surveillance site in Svay Rieng Province. We applied WHO Manual‐derived methods to count respiratory hospitalizations at all hospitals within the catchment area, where 95% of the sentinel site case‐patients resided. We used HAS methods to adjust the district‐level population denominator for the sentinel site and calculated the incidence rate of influenza‐associated SARI by dividing the number of influenza‐positive SARI infections by the adjusted population denominator and multiplying by 100 000. We extrapolated the rate to the provincial population to derive a case count for 2015. We evaluated data sources, detailed steps of implementation, and identified lessons learned.

**Results:**

We estimated an adjusted influenza‐associated 2015 SARI rate of 13.5/100 000 persons for the catchment area of Svay Rieng Hospital and 77 influenza‐associated SARI cases in Svay Rieng Province after extrapolation.

**Conclusions:**

Methods detailed in the WHO Manual and operationalized successfully in Cambodia can be used in other settings to estimate rates of influenza‐associated SARI.

## 1 INTRODUCTION

Influenza is an acute viral infection and a significant contributor to global morbidity^1^, ^2^, ^3^ and mortality.^4^, ^5^ While the burden of influenza has been established in some countries, it is not well understood in many others, particularly lower‐middle‐income countries such as Cambodia.^6^ Producing national estimates of influenza‐associated morbidity and mortality and understanding influenza's impact on health systems are key deliverables in the Pandemic Influenza Preparedness Partnership Contribution Implementation Plan 2013‐2016,^7^ established to implement a global approach to pandemic influenza preparedness and response.

The World Health Organization's (WHO) global standards for influenza surveillance are used by many countries.^8^, ^9^, ^10^, ^11^ Severe acute respiratory infection (SARI) surveillance intends to capture hospitalized influenza‐associated severe respiratory illness cases and uses a case definition—either recommended by WHO or country‐specific—to identify severe presentations of influenza‐associated respiratory disease.^12^ The Cambodia‐based Centers for Disease Control and Prevention and Ministry of Health's (C‐CDC/MOH) SARI sentinel surveillance system defines a SARI case as a hospitalized patient with measured temperature ≥38°C or self‐reported fever with symptoms of cough or sore throat, and shortness of breath or difficulty breathing within 10 days of hospital admission. The WHO‐recommended case definition does not require shortness of breath and/or difficulty breathing.^11^ Cambodia has conducted influenza surveillance since 2009, including both outpatient influenza‐like illness surveillance and hospitalized SARI surveillance, and enrolls all patients meeting the Cambodia SARI case definition at eight sentinel surveillance sites. Surveillance has demonstrated that influenza viruses commonly circulate during the rainy season (June‐November) in Cambodia each year.^13^, ^14^ Between 2009 and 2014, influenza circulation peaked between August and November; influenza positivity among SARI cases fluctuated from 12% in 2009 to 3% in 2014.^15^

To better understand the impact of influenza on the Cambodia population and inform possible future vaccine policy, health officials are exploring best practices to estimate the burden of influenza‐associated SARI in the country.

While several methods have been employed by other countries,^1^, ^2^, ^16^, ^17^, ^18^, ^19^, ^20^ rate calculations are not always possible because the appropriate population count is not easily ascertained. Historically, healthcare utilization surveys (HUS) have been used to determine appropriate population denominators by estimating the catchment area for a hospital through an understanding of the population's healthcare‐seeking behavior.^21^, ^22^, ^23^ However, this method requires significant financial and staffing resources, involving house‐to‐house surveys in a random sample of representative households to inquire about healthcare facility use.^6^ To assist countries in estimating influenza‐associated burden who are unable to undertake an HUS, WHO developed *A Manual for Estimating Disease Burden Associated With Seasonal Influenza* (WHO Manual),^6^ which outlines an alternative method—a hospital admission survey (HAS)* —for estimating population denominators using existing data sources. We present an application in one province of Cambodia using HAS methods to estimate the population of the catchment area and calculate the burden of influenza‐associated SARI. We detail our methods and provide results and lessons learned.

## 2 METHODS

### 2.1 Overview of the WHO Manual

The WHO Manual contains guidance for identifying and selecting data sources, reviewing available data for quality and relevance, determining the geographic area where ≥80% of the sentinel site's case‐patients reside (catchment area), and using HAS methods to estimate an appropriate population denominator. It outlines how to use the HAS, catchment population, and SARI sentinel surveillance data to calculate the burden of influenza‐associated SARI.

### 2.2 Data sources

Existing surveillance, hospital, and population data were evaluated for suitability to estimate the rate of influenza‐associated SARI.

#### 2.2.1 Sentinel surveillance at Svay Rieng Provincial Hospital (SRPH)

**Figure 1:**
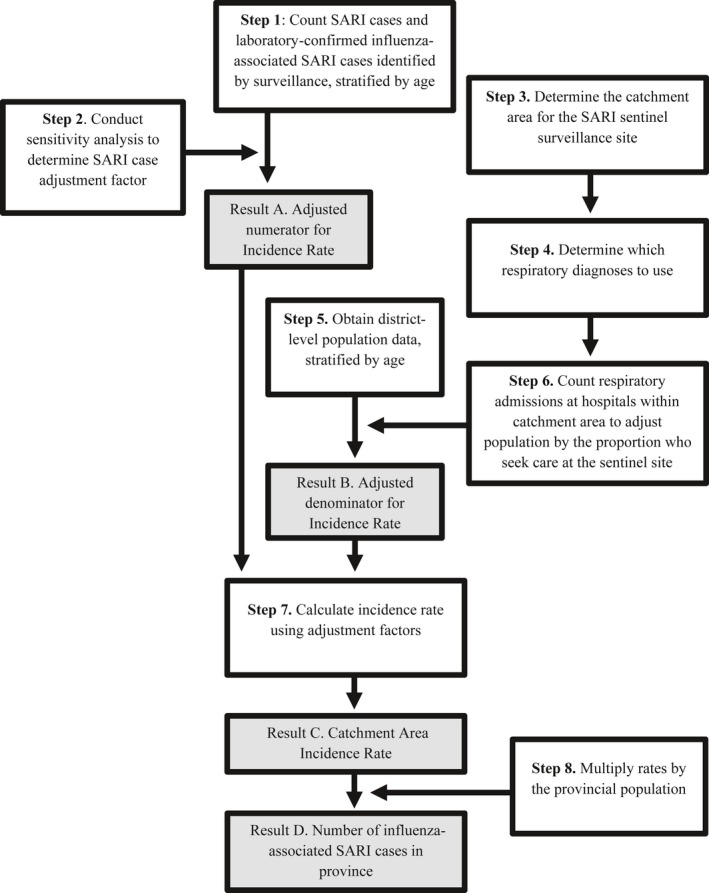
Overview of methodology to calculate annual number of influenza‐associated severe acute respiratory infections in a province

**Figure 2:**
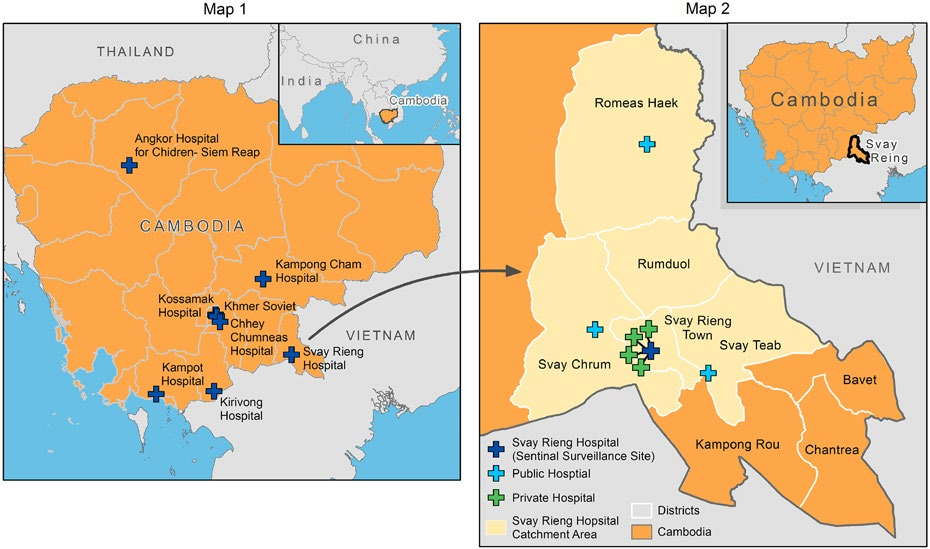
**Map 1.** Sentinel sites for severe acute respiratory infection surveillance in Cambodia.**Map 2.** Svay Rieng Province, Cambodia and Svay Rieng Provincial Hospital catchment area

We used data previously collected via C‐CDC/MOH's SARI sentinel surveillance at SRPH to count the number of SARI cases and influenza‐associated SARI cases in Svay Rieng Province in 2015 (Figure 1: Step 1). SRPH is located in Svay Rieng Province in southeast Cambodia, bordering Vietnam (Figure 2). It is the only public referral hospital in the province and admits patients of all ages. Sentinel surveillance is the method recommended by the WHO Manual for performing influenza surveillance. SRPH became a sentinel surveillance site in 2014 as part of an effort to expand and increase the geographic diversity of SARI surveillance in Cambodia.

#### 2.2.2 Hospital medical records at SRPH

We used medical chart records at the sentinel surveillance site (SRPH) to assess for under‐reporting of SARI cases (sensitivity analysis) to the SARI surveillance system (Figure 1: Step 2).

#### 2.2.3 Counts of respiratory hospitalizations within the catchment area

We reviewed admission and discharge registers at all public and private hospitals within the catchment area of SRPH to count the number of hospitalizations for any respiratory illness by age group for each hospital (Figure 1: Steps 3, 4, 6). Registers capture basic demographic and hospitalization data on each patient admitted to the hospital using a line‐list format.

#### 2.2.4 Svay Rieng province health center population data

Svay Rieng Province obtains population counts from each health center within the province each year (Figure 1: Step 5). These counts are reported to each operational district's health center by the village leaders and are collated and reported to the provincial health department. These counts provide district‐level population data by age group; however, collection practices reportedly differ by district.

#### 2.2.5 Census‐collected population data

Cambodia last conducted a census in 2008 and uses birth, death, and migration rates to extrapolate population estimates by age group, gender, and province.^24^ We did not use this data source as it did not contain district‐level population estimates, a necessary stratification for HAS methods.

#### 2.2.6 Nationally collected health statistics

Cambodia's MOH collects data on all hospitalizations at public hospitals and national private hospitals within Cambodia. Data are stratified by age group, gender, and primary diagnosis. We did not include this data source in our calculations because validation to determine the accuracy of the data collected was not completed. Data collected through an HAS could be used to validate this data source for a future national‐level burden estimation.

### 2.3 Overview of burden calculation

To estimate the rate of influenza‐associated SARI in 2015, we applied methods from the WHO Manual to abstract SARI sentinel surveillance data (Figure 1: Step 1) and conducted a sensitivity analysis of the data with chart review of selected weeks (Figure 1: Step 2). We counted respiratory hospitalizations at all hospitals within the sentinel site's catchment area, applying HAS methods from the WHO Manual (Figure 1: Steps 3, 4, 6). We multiplied the population of the catchment area by the percent of respiratory admissions that were hospitalized at the sentinel site to obtain a population count adjusted for those who sought care at the sentinel site (Figure 1: Step 6). We then divided the number of influenza‐associated SARI cases per age group by the adjusted population denominator (Figure 1: Step 7) and multiplied by 100 000 to obtain 2015 incidence rates of influenza‐associated hospitalizations (Figure 1: Step 8). The steps to calculate estimates are further outlined below.

Step 1. We counted SARI cases and influenza‐associated SARI cases identified by the sentinel surveillance system at SRPH in 2015, stratified by age. The WHO Manual recommends at least 1 year of SARI surveillance; we selected 2015, as that was the first year SRPH collected district‐level population data on SARI cases. We followed the WHO‐recommended age groups for influenza surveillance reporting for those ≥15 years, specifically: 15‐49 years, 50‐64 years, and ≥65 years.^11^ For those <15 years of age, we used age groups that corresponded with available population data: <1 year and 1‐14 years. A SARI case at SRPH was defined according to the C‐CDC/MOH case definition. SRPH clinicians attempted to enroll all hospitalized patients meeting the SARI case definition from all medical, non‐obstetric wards. Nasopharyngeal swabs, oropharyngeal swabs, or sputum samples were taken from enrolled case‐patients and were tested for influenza viruses at the National Institute for Public Health (NIPH) in Phnom Penh using real‐time reverse transcription polymerase chain reaction (rRT‐PCR).

Step 2. We conducted a sensitivity analysis at SRPH to evaluate SARI case ascertainment and to determine whether adjustments were needed to account for missing information. We compared SARI case‐patients identified through the surveillance system during selected weeks (Epi Weeks 4, 24, 27, 30, 37, and 40)† to hospitalized patients identified by chart review during the same time period. From each medical record, we collected age, district of residence, admission and discharge dates, discharge diagnosis, respiratory symptoms (fever, cough, sore throat, difficulty breathing), and illness onset date. We classified each patient as either a SARI case or not using both C‐CDC/MOH and WHO SARI case definitions. We divided the number of cases identified through chart review by the number reported through the surveillance system and applied the calculated ratio as an adjustment to the numerator in our burden calculation (Figure 1: Result A).

Step 3. We identified the catchment area, which is the geographic area where most case‐patients seeking care at the sentinel site resided. The WHO Manual recommends that the catchment area for a sentinel site should include the administrative divisions where ≥80% of the SARI case‐patients reside for the site. We used address data from the SARI surveillance system to determine the districts within the province where the majority of SARI case‐patients lived.

Step 4. We evaluated respiratory diagnoses to capture the most applicable hospital admissions. While the WHO Manual recommends using a diagnosis of pneumonia to determine the number of respiratory hospital admissions, we were concerned that using one specific definition might result in missing some SARI‐related admissions. None of the hospitals within the catchment area used International Classification of Diseases (ICD) coding for admission and discharge diagnoses, so we evaluated a comprehensive list (Table A1 in Appendix) of free‐text diagnoses and compared them to recorded discharge diagnoses from both SARI case definitions to determine the most appropriate ones for future use.

Step 5. We obtained district‐level population data stratified by age. To calculate the influenza‐associated SARI rate, we used age‐specific population data for the catchment area of our sentinel site. While we identified two sources of population data, we used the Svay Rieng Province Health Center Population data because district‐level population data were necessary to match the population denominator to the catchment area.

Step 6. With our HAS, we counted respiratory hospitalizations at all hospitals within the catchment area to determine the proportion of respiratory hospitalizations at the sentinel site, relative to all respiratory hospitalizations. We manually reviewed hospital admission and discharge registers to determine the number of respiratory admissions using the predetermined list of respiratory diagnoses at each hospital within the catchment area. We recorded age, gender, district of residence, admission and discharge diagnoses, symptom onset date, and the outcome of each patient that lived within the catchment area and had at least one respiratory diagnosis associated with their hospitalization. The count of respiratory hospitalizations, stratified by age, at the sentinel site was divided by the count of total respiratory admissions at all hospitals within the catchment area for that age group. The resulting proportional number of respiratory admissions at the sentinel site was multiplied by the population of the catchment area to create an adjusted population denominator by age group for the incidence rate calculation (Figure 1: Result B).

Step 7. We calculated the influenza‐associated SARI rate using adjustment factors (Figure 1: Result C).

- *Adjusted population denominator formula*: population in catchment area by age group × percentage of admissions at sentinel site by age group
- *Incidence rate formula*: (Influenza‐associated SARI cases/adjusted population denominator) × 100 000

Step 8. We multiplied resulting rates by the age‐specific provincial population data to obtain a provincial estimate of influenza‐associated SARI cases for SRPH in 2015 (Figure 1: Result D).

## 3 RESULTS

### 3.1 SARI cases and catchment area

During 2015, 214 SARI case‐patients at SRPH were identified and enrolled in the surveillance system. Svay Rieng Province has one town and seven districts. We included Svay Rieng town and four districts, capturing 95% (203/214) of SARI case‐patients in the catchment area (Figure 2). Of those, 8% (n = 17) of 203 specimens tested were positive for influenza (Table 1, Figure A1 in Appendix). We identified all medical facilities located within the catchment area of SRPH, which included three public district hospitals and four private admitting facilities (Figure 2).

**Table 1:** Severe acute respiratory infections (SARI) and influenza‐associated SARI cases by age group—Svay Rieng Provincial Hospital catchment area, 2015

Age group	SARI cases	Influenza‐associated SARI cases (95% CI)	Percent positive for influenza
Birth to <1 y	8	0 (0‐0)	0
1‐14 y	24	4 (0‐8)	16.7
15‐49 y	44	4 (0‐8)	9.1
50‐64 y	61	4 (0‐8)	6.6
≥65 y	66	5 (1‐9)	7.6
Total	203	17 (9‐25)	8.4

### 3.2 Sensitivity analysis of SARI case ascertainment

We reviewed 475 medical charts from six weeks at SRPH in 2015 (Epi Weeks 4, 24, 27, 30, 37, and 40) and identified 216 with evidence of fever. Of those, we identified 41 (19%) patients who met the C‐CDC/MOH SARI case definition of a hospitalized patient with measured temperature ≥38°C or self‐reported fever with symptoms of cough or sore throat, and shortness of breath or difficulty breathing within 10 days of hospital admission. Using the WHO case definition, which does not require evidence of difficulty breathing, we identified 101 SARI case‐patients. During those same 6 weeks, the surveillance system identified 50 SARI case‐patients. The ratio of cases identified during the chart review compared to those identified through the surveillance system was 41/50 or 0.8 using the C‐CDC/MOH SARI case definition; using the WHO case definition, the case ratio was 101/50 or 2.0.

### 3.3 Respiratory diagnoses identified through chart review

Of the 41 patients who met the C‐CDC/MOH SARI case definition, 40 (98%) had an admission or discharge diagnosis consistent with a respiratory illness. The most frequently found diagnoses were pneumonia (16, 39%), pharyngitis (11, 27%), bronchiolitis (6, 15%), and asthma (6, 15%). Of the 101 hospital admissions that met the WHO SARI case definition, 90 (89%) had an admission or discharge diagnosis consistent with a respiratory illness. Pharyngitis (47, 47%); pneumonia, including severe pneumonia and bronchopneumonia (20, 19%); bronchiolitis (9, 9%); and asthma (6, 6%) were the most common diagnoses. Of the 11 patients without a respiratory diagnosis, the following diagnoses were observed: neonatal infection (5), hyperthermia (1), dysentery (1), malnutrition (1), enteritis (1), dengue (1), and intoxication (1).

### 3.4 Population data

Using Svay Rieng Province Health Center Population Data, the Svay Rieng Province population in 2015 was 596 539 and the population within the catchment area was 487 489 (Table 2a).

### 3.5 Proportional respiratory admissions

**Figure 3:**
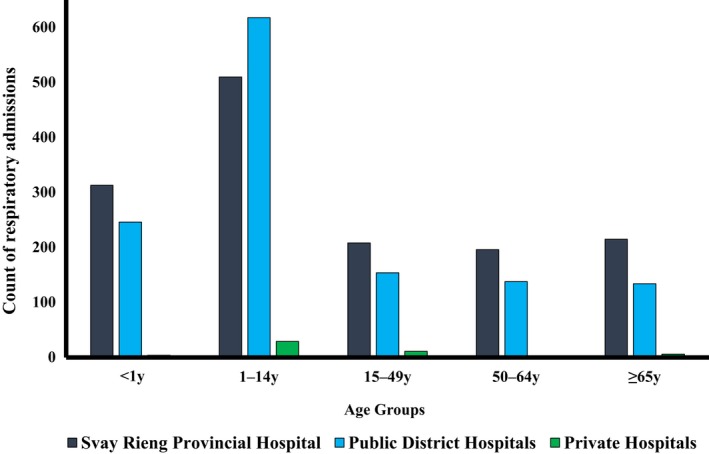
Count of respiratory admissions in 2015 by hospital type and age group, Svay Rieng Provincial Hospital catchment area

Of the 2786 respiratory hospital admissions within the catchment area in 2015, 46% (1290) were from public district hospitals, 2% (54) from private admitting facilities, and 52% (1442) from SRPH, with differences by age (Figure 3).

### 3.6 Influenza‐associated SARI rate in the SRPH catchment area

We calculated the influenza‐associated SARI rate using both SARI case definitions. Using the C‐CDC/MOH case definition, we estimated an influenza‐associated SARI rate for all ages of 6.7/100 000 persons in 2015 within the SRPH catchment population (Table 2). After adjusting by a factor of 2.0 for possible decreased case ascertainment using the C‐CDC/MOH case definition compared to the WHO case definition, we estimated an influenza‐associated SARI rate of 13.5/100 000 persons (Table 2b).

**Table 2:** (a) Population counts, percent influenza‐associated severe acute respiratory infections (SARI) at SARI sentinel site, and adjusted population counts by age group—Svay Rieng Province, 2015. (b) Influenza‐associated severe acute respiratory infections (SARI) by age group and incidence rate adjustment method—Svay Rieng Province, 2015

(a)				
Population and admissions data				
Age group	Population in catchment area	Percent admissions at SRPH (%)^a^	Adjusted catchment area population count^b^	Total Svay Rieng population
Birth to <1 y	9649	56	5403	11 807
1‐14 y	129 245	44	56 868	158 158
15‐49 y	266 248	56	149 099	325 806
50‐64 y	57 531	58	33 368	70 401
≥65 y	24 816	60	14 890	30 367
Total	487 489	52	253 494	596 539

(b)						
Age group	SARI cases and incidence rate using unadjusted SARI case count reported through surveillance			SARI cases and incidence rate using SARI case count reported through surveillance, adjusted by sensitivity analysis using the WHO case definition^c^		
	Influenza‐associated SARI case‐patients in catchment area (95% CI)	Incidence rate for SRPH (per 100 000)^d^ (95% CI)	Total case‐patients in Svay Rieng Province^e^ (95% CI)	Influenza‐associated SARI case‐patients in catchment area (95% CI)	Incidence rate for SRPH (per 100 000)^d^ (95% CI)	Total case‐patients in Svay Rieng Province^e^ (95% CI)
Birth to <1 y	0 (0‐0)	0 (0‐0)	0 (0‐0)	0 (0‐0)	0 (0‐0)	0 (0‐0)
1‐14 y	4 (0‐8)	7.0 (0‐14.1)	11 (0‐22)	8 (2‐14)	14.2 (4.3‐24.1)	22 (7‐38)
15‐49 y	4 (0‐8)	2.7 (0‐5.3)	9 (0‐17)	8 (2‐14)	5.4 (1.7‐9.2)	18 (5‐30)
50‐64 y	4 (0‐8)	12 (0.1‐23.9)	8 (0‐17)	8 (2‐14)	24.2 (7.4‐41.0)	17 (5‐29)
≥65 y	5 (1‐9)	33.6 (3.9‐63.3)	10 (1‐ 19)	10 (4‐16)	67.8 (25.8‐109.9)	20 (8‐33)
Total	17 (9‐25)	6.7 (3.5‐9.9)	38 (20‐57)^f^	34 (23‐46)	13.5 (9.0‐18.1)	77 (51‐104)^f^

a Svay Rieng Provincial Hospital.

b Population in catchment area multiplied by percent admissions at SRPH.

c History of fever or measured fever of ≥38°C and cough and onset within the last 10 d and requires hospitalization^13^.

d Influenza‐associated SARI cases divided by the adjusted catchment area population denominator.

e (Incidence rate for SRPH multiplied by the total Svay Rieng Population) divided by 100 000.

f Column total.

### 3.7 Influenza‐associated SARI cases in Svay Rieng Province

Using the unadjusted influenza‐associated SARI rate, we estimated 38 influenza‐associated SARI cases in Svay Rieng Province in 2015 after extrapolating the rate to the provincial population (n = 596 539). By adjusting for possible under‐ascertainment of cases by using the WHO case definition and applying the resulting catchment area hospitalization rate to the provincial population, we estimated 77 influenza‐associated SARI cases in Svay Rieng Province in 2015 (Table 2b).

## 4 DISCUSSION AND LESSONS LEARNED

By applying methods outlined in the WHO's *A Manual for Estimating Disease Burden Associated With Seasonal Influenza* to the available data sources and context of Svay Rieng Province in Cambodia, we derived the first provincial‐level estimate of influenza‐associated SARI in Cambodia. This information may be used by the province to guide prevention and control practices and provides the first step toward a national estimate of influenza burden in Cambodia.

Many factors influence estimates, including seasonal variation in influenza activity, healthcare‐seeking behavior, and underlying health status of the population, which make direct comparisons difficult to interpret. Our estimated rate was similar to estimates from one country^16^ but lower than other studies,^25^, ^26^ despite a similar influenza‐positivity proportion among enrolled SARI cases. A study in Oman using case definitions that differed from the case definition used in Cambodia found incidence rates similar to ours, with a range of 0.5‐15.4 per 100 000 persons in the years 2008 to 2013.^16^ A 2014 study in the Philippines, using the same case definition as C‐CDC/MOH, found an influenza‐associated SARI incidence rate of 100 per 100 000 persons,^26^ a sevenfold higher estimate than ours. A 2013 study in Kenya defined a SARI case as a hospitalized patient with cough, difficulty breathing, or chest pain during the previous 14 days, or a hospitalized patient with fever with cough or shortness of breath or difficulty breathing. They estimated an incidence rate of 290‐470 cases per 100 000 persons for patients aged less than five years and 20 per 100 000 persons for those aged >5 years.^25^ Differences in the case definition used for enrollment could account for some of the variation in estimates between countries, in addition to factors mentioned previously.

Our study had several limitations. We found a lower rate of influenza‐associated SARI than we expected. Cambodia's case definition requires that the patient have difficulty breathing. This definition is more specific than the currently recommended WHO case definition and may have limited case enrollment; however, even after adjusting for possible low case ascertainment using C‐CDC/MOH case definition, our estimate was still low. SRPH is a relatively new sentinel site (initiated in 2014) and surveillance implementation challenges may have resulted in identification and enrollment of fewer case‐patients than met the case definition. We may also have missed a large fraction of the population that either chose not to seek care for their illness (ie, community burden of disease) or sought care outside of the province. Our study could not account for these factors. Our estimate was for one province of Cambodia, which may not be representative of Cambodia; this initial estimate was not intended to provide a national estimate. Future work to generate a national estimate is needed to understand the burden of influenza‐associated SARI in Cambodia.

The HAS method described by the WHO Manual^6^ has several advantages. First, it is a relatively simple method that can be used in other settings where hospitalization and population records are available. We successfully conducted data collection in 7 days with 12 data collectors and six supervisors using existing population data, existing paper‐based medical records, and hospital admission and discharge registers as data sources. Second, it employs established laboratory‐based surveillance systems for influenza and encourages the evaluation and strengthening of these systems to ensure the collection of high‐quality data. Third, it allows health officials to better understand healthcare‐seeking behavior for respiratory illnesses within their jurisdiction.

Each data source used in our estimate presented challenges for use and interpretation. By applying the WHO Manual to Svay Rieng Province, we learned lessons that may be useful to other settings where the WHO Manual is being considered.

While the intent of a burden study is not to review and evaluate the surveillance system, such review may inform projects that use the surveillance system and may ensure the quality and reliability of burden estimates, future surveillance methods, and reporting. For this study, we used a sensitivity analysis through a retrospective chart review to evaluate the application of the case definition to hospitalized patients at SRPH and adjusted our estimated number of case‐patients accordingly. The validity of the sensitivity analysis is dependent on the quality of the patient's medical record and the accurate recording of symptoms such as fever, cough, sore throat, and difficulty breathing.

In our study, we also evaluated hospital records to allow us to count the number of respiratory hospitalizations with the predetermined diagnoses and to determine the catchment population. The minimum patient record required for an HAS is a hospitalization register that includes admission and/or discharge diagnoses, date, age, gender, district, diagnoses, and patient outcome. Hospital registers provide the ability to count respiratory hospitalizations. Such records must be available for all hospitals in the catchment area; if electronic or paper patient records are not available or organized for a review, an HAS will not be possible.

We learned that there may be different sources of hospitalization information that could be identified and reviewed. During our study, we learned that the MOH collects hospitalization data from public and large private hospitals. Other countries may also collect national‐level health data or subnational hospitalization data which may be evaluated and used to estimate burden. When reviewing data sources for suitability, the following should be considered: types of respiratory diagnoses; potential missing data (hospitals, diagnoses, high‐risk groups); age and gender data; protocols and application of reporting; differences between hospitals; and any previous evaluation or validation of data sources.

We learned that it is important to review the relevant respiratory diagnoses when conducting an HAS. Our findings suggest that the diagnoses list for HAS data collection should be expanded beyond pneumonia. In Cambodia, it could be limited to small set of diagnoses: pharyngitis, pneumonia, bronchiolitis, asthma, and bronchitis. However, commonly used diagnoses may vary greatly by country and hospital; therefore, an evaluation to optimize the list of included diagnoses is recommended before beginning an HAS.

Another lesson learned was the importance of identifying and evaluating all possible data sources, including population data. The HAS methodology requires reliable and accessible population data for relatively small administrative units. The availability and quality of these data must be evaluated before deciding to conduct an HAS. We identified two sources of population data, one of which had data stratified by district, which we were able to use for our provincial estimate.

## 5 CONCLUSION

Using HAS methods derived from the WHO Manual and several pre‐existing data sources, we estimated an influenza‐associated SARI rate of 6.7/100 000 persons for the catchment area of Svay Rieng Hospital; after adjusting for possible decreased case ascertainment using the C‐CDC/MOH case definition, we estimated a rate of 13.5/100 000 persons. We evaluated data sources, detailed steps of implementation, and identified lessons learned. A careful review of all available data sources before beginning data collection will improve the feasibility of the study and quality of the data collected. Health officials may find our operationalization of the WHO Manual methods and detailed steps helpful when working toward similar estimates in similar contexts.

## CONFLICTS OF INTEREST

We declare that we have no conflict of interests.

## DISCLAIMER

The findings and conclusions in this report are those of the authors and do not necessarily represent the official position of the Centers for Disease Control and Prevention.

## Notes

*In Cambodia, we used the term “Hospital Admission Review” instead of “Hospital Admission Survey” in accordance with surveillance and public health practice terminology requested by the Ministry of Health, Communicable Disease Control Department in Cambodia.

†Epi weeks were defined as: Week 04: 21‐27 Jan, Week 24: 10‐16 Jun, Week 27: 01‐07 Jul, Week 30: 22‐28 Jul, Week 37: 09‐15 Sep, Week 40: 30 Sep‐06 Oct, 2015.

## APPENDIX 1

### 1.1

**Table A1** Full list of used and analyzed free‐text respiratory admission and discharge diagnoses for inclusion in hospital admission survey in Cambodia

**Table:**

Pneumonia
Severe pneumonia
Bronchopneumonia
Broncho‐asthma
Bronchitis
Bronchiolitis
Respiratory illness
Pneumopathy
Pulmonary tuberculosis (TB)
Asthma
Pharyngitis
Rhino‐pharyngitis
Tonsillitis
Laryngitis
Lung abscess/empyema

### 1.2

**Figure A1** Influenza virus positivity among SARI case‐patients by month, Svay Rieng Provincial Hospital, Cambodia, 2015
